# Effects of Total Sulfur Amino Acids on Growth Performance, Immunity, and Meat Yield in Broilers Fed Diets With and Without Antibiotics

**DOI:** 10.3389/fvets.2022.903901

**Published:** 2022-06-02

**Authors:** Pratima Acharya Adhikari, Fernanda Lima de Souza Castro, Guanchen Liu, Woo Kyun Kim

**Affiliations:** ^1^Department of Poultry Science, Mississippi State University, Starkville, MS, United States; ^2^Department of Poultry Science, University of Georgia, Athens, GA, United States

**Keywords:** antibiotic as growth promoter, broiler, carcass yield, chicks, growth performance, sulfur amino acids

## Abstract

Two experiments were conducted to evaluate the effects of digestible sulfur amino acids (SAA) on performance, carcass yield, immunity, and amino acid transporters in broilers fed diets with or without an antibiotic growth promoter (AGP). In experiment 1, a total of 250 1-day-old Cobb500 male chicks were assigned to battery cages with two levels of AGP (0 and 0.05% bacitracin) and five levels of SAA (0.7, 0.8, 0.9, 1.0, and 1.1%) for 21 d. In experiment 2, a total of 900 1-day-old Cobb500 male chicks were assigned to floor pens with two levels of AGP and three levels of SAA for the starter (0.7, 0.8, and 0.9%) or finisher phase (0.52, 0.62, and 0.72%) for 42 d. In experiment 1, from 0 to 7 d, the body weight gain (BWG) was the lowest for birds fed 0.7% SAA. The AGP significantly decreased the feed conversion ratio (FCR), and birds fed 0.9 and 1.1% SAA had significantly lower FCR than 0.7% SAA. From 8 to 14 d, for the AGP-fed birds, the lowest BWG was observed in the 0.7% SAA group. In birds not fed AGP, birds fed 0.8% SAA had higher BWG than 0.7 and 1.1% SAA. Birds fed 0.7% SAA diet had lower feed intake (FI) than 0.8% SAA and higher FCR than 0.8, 0.9, and 1.0% SAA. In experiment 2, from 0 to 21 d, the lowest BWG and the highest FCR were observed in birds fed 0.7% SAA, whereas birds fed 0.9% SAA had the highest BWG and lowest FCR. From 22 to 42 d, FCR was lower for birds fed AGP, and for birds fed 0.72%. Interactions between the factors were found for FI and BWG. The whole thigh and wing weights were the highest for 0.62% SAA, and the pectoralis major weight was higher for birds fed 0.62% SAA than those fed 0.52% SAA. There was an interaction between SAA and AGP for Lat1 (large neutral amino acid transporter) expression, and AGP-fed birds had higher expression of ileal interleukin 1β (Il−1β gene). The interleukin 10 (Il-10) expression was upregulated in the ileum. There was an interaction between factors for sodium-dependent neutral amino acid transporter B [0] AT1 (SLC6A19) expression. The results suggested that both AGP and SAA supplementation would affect the growth performance of the broilers.

## Introduction

Commercial broiler diets containing soybean meal as the primary protein source are deficient in sulfur amino acids (SAA), and the synthetic supplementation of methionine (Met) is a common practice (1, 2). The SAA include Met and cysteine (Cys), which are used by broilers in several ways such as feather development, improvement of growth performance, and production of antibodies (3). Methionine has an immune regulatory action (4, 5), playing a major role in humoral and cellular immune responses in poultry (6, 7). However, a level higher than the optimal growth level is required for improving the immune response (3).

For about 50 years, broiler diets have been fortified with antibiotics as growth promoters (AGPs) to reduce intestinal microflora and improve growth (8, 9). It was proposed that the AGPs could promote growth performance by reducing sub-clinical infections, and therefore, reducing the production of growth-depressing metabolites and nutrient utilization of the microbiome and resulting in the enhanced nutrient uptake of the host animals. (10). Previous research has also shown that the effectiveness of AGPs could be affected by the environment. When broilers are raised in battery cages, the effects of AGPs such as bacitracin, avilamycin, and monensin are minimal, whereas such effects are pronounced in floor pens. Such difference was possibly due to the different extents of pathogen infections as well as different microbial communities (11, 12). More importantly, the use of such non-therapeutic AGPs in the food of animals has been pointed out as one of the causes of antibiotic resistance in the animals and the spreading of such resistance from animals to humans is posing a serious threat to public health (13–15). As a result, AGPs have been banned in Europe and other parts of the world. In the United States, consumer pressure has become one of the main reasons behind raising chickens without AGPs (14, 16). There is a challenge to maintain optimal animal production by withdrawing AGPs in feed without simultaneously compromising growth performance and feed efficiency. Alternative products to AGPs, such as probiotics, prebiotics, and organic acids have been used for improving animal health and production (17). Furthermore, nutritional approaches, such as supplementing extra SAA with immune response regulatory functions, could also possess the potential to maintain the performance of AGPs' withdrawal broilers.

The inclusion of digestible SAA at 0.88–0.90% in the broiler starter diets supplemented with AGPs has been already recommended (18). Previous studies had shown that withdrawing the AGPs in the broiler diets can lead to decreased growth performance as well as affected intestinal microflora (19, 20) The optimal dietary SAA levels change with production goal, like breast meat yield or growth performance (21). It would be plausible to assume that withdrawing AGPs in diets can change the optimal SAA levels for the broilers. Few studies have been done to study the effects of AGPs withdrawal in diets containing different levels of SAA on the growth performance of broilers. Additionally, withdrawal of AGPs could affect the immune response of the broilers (19, 22), as previously described, methionine plays a role in immune responses regulation, supplementing extra methionine might reverse the adverse effect caused by AGPs withdrawal. Moreover, studies involving the intestinal immune system and transporter genes in broilers fed diets supplemented with both SAA and AGPs are still lacking in the literature. Therefore, we have conducted two studies to evaluate the effects of different levels of SAA on growth performance, carcass yield as well as expression of immune and Met transporter genes in broiler chickens, fed diets with or without AGPs.

## Materials and Methods

The experimental protocol was reviewed and approved by the University of Georgia Institutional Animal Care and Use Committee (A2016 04–017).

### Experiment 1

#### Animals, Diets, and Experimental Design

The experiment was conducted at the University of Georgia, Poultry Research Center (Athens, GA), under the approval of the Institutional Animal Care and Use Committee (IACUC) (A2016 04–017). A total of 250 1-day-old Cobb-500 male broiler chicks were randomly allocated to ten dietary treatments in a 2x5 factorial arrangement, with two levels of AGP (0 and 0.05% bacitracin) and five levels of SAA (0.7, 0.8, 0.9, 1.0, and 1.1%) as the main effects. Each treatment had five replicates of five chicks each. The levels of SAA were obtained by supplementing DL-Met to the basal diet that contained 0.7% SAA. DL-Met was added at 0, 0.1, 0.2, 0.3, and 0.4% into the basal diet to make the treatment diets containing 0.7, 0.8, 0.9, 1.0, and 1.1% SAA, respectively. The chicks were raised in batteries (Petersime Incubator Co., Gettysburg, OH) with controlled electrical heating, lighting, and trough-type feeders and waterers for 21 d. The lighting program started with 23 h of light from 0 to 7 d, 22 h of light from 8 to 14 d, and 20 h of light from 15 to 21 d. All chickens received a broiler starter diet containing 3,200 kcal/kg of metabolizable energy (ME) and 23% crude protein (CP) and ad libitum water. All other essential nutrients were provided either to meet or exceed starter broiler chicks' requirements except for SAA (Table 1).

**Table 1:** Diet composition and nutritional values (experiment 1).

**Ingredients^a^**	**Amount (%)**
Corn, grain	51.70
Soybean meal (48%)	36.97
Poultry fat	6.60
Defluorated phosphate	2.00
L-Lysine HCL	0.94
Limestone	0.66
Common salt	0.40
Chromic oxide	0.30
Vitamin premix^b^	0.25
Mineral premix^c^	0.08
Coccidiostat	0.05
Antibiotics^d^	0.05
Nutrient content	
ME (kcal/kg)^e^	3200.00
CP (%)^e^	23.00
SAA (%)^e^	0.70

a *DL-methionine was added in first 5 diets at the rate of 0, 0.04, 0.08, 0.12 and 0.16% in basal diet and the remaining 5 diets had similar formulation but without AGP.*

b *Vitamin premix provided the following (per kg of diet): thiamin mononitrate, 2.4 mg; nicotinic acid, 44 mg; riboflavin, 4.4 mg; D-Ca pantothenate, 12 mg; vitamin B12 (cobalamin), 12.0 g; pyridoxine HCl, 4.7 mg; D-biotin, 0.11 mg; folic acid, 5.5 mg; menadione sodium bisulfite complex, 3.34 mg; choline chloride, 220 mg; cholecalciferol, 27.5 g; transretinyl acetate, 1,892 g; α tocopheryl acetate, 11 mg; ethoxyquin, 125 mg.*

c *Mineral premix provided the following (per kg of diet): manganese (MnSO4.H2O), 60 mg; iron (FeSO4.7H2O), 30 mg; zinc (ZnO), 50 mg; copper (CuSO4.5H2O), 5 mg; iodine (ethylene diamine dihydroiodide), 0.15 mg; selenium (NaSe03), 0.3 mg.*

d *BMD-50 (Bacitracin Methylene Disalicylate) was added as an antibiotic growth promoter.*

e *ME, metabolizable energy; CP, crude protein; SAA, sulfur amino acids.*

#### Measurement of Growth Performance Data

The broilers and feed were weighed collectively by cage on d 0, 7, 14, and 21 using a standard weighing scale (Mettler Toledo Co.). Feed additions were weighed and recorded. The bodyweight gain (BWG) was calculated by subtracting initial bird weight from final weight, and weekly feed intake (FI) was also calculated by subtracting feed amount consumed in the respective weighing periods. Feed conversion ratio (FCR) was calculated as the ratio of FI to BWG. The dead chicks were removed and weighed daily to calculate mortality and adjusted growth performance data.

### Experiment 2

#### Animals, Diets, and Experimental Design

The experiment was conducted at the University of Georgia, Poultry Research Center (Athens, GA), under the approval of the Institutional Animal Care and Use Committee (IACUC) (A2016 04–017). A total of 900 1-day-old Cobb500 broiler chicks were randomly distributed to six dietary treatments in a 2×3 factorial arrangement, with six replicates of 25 birds each. The main effects were two levels of AGP (0 and 0.05% bacitracin) and 3 levels of SAA either for the starter (0.7, 0.8, and 0.9%) or finisher phase (0.52, 0.62, and 0.72%). The chicks were housed in floor pens equipped with nipple water drinkers (Ziggity Systems Inc., Middlebury, IN), feeders, and wood shavings. The lighting schedule was followed according to Cobb (18). The chicks were fed a corn–soybean starter diet with 21% CP and 3,100 kcal/kg of ME from 0 to 21 d. After d 21, the chicks received a finisher diet with 18% CP and 3,100 kcal/kg of ME until d 42. Feed and water were provided *ad libitum*. All other essential nutrients were provided to meet the NRC starter and finisher broiler requirements (23) except for SAA (Table 2). For the starter phase, DL-Met was added at 0.02, 0.12, and 0.22% into the basal diet containing 0.68% Met to make dietary treatments containing 0.7, 0.8, and 0.9% SAA, respectively. For the finisher phase, 0, 0.01 and 0.12% DL-Met were added to make dietary treatments containing 0.52, 0.62, and 0.72% SAA, respectively.

**Table 2:** Diet composition and nutritional values (experiment 2).

**Ingredients and composition, %**	**Starter diet^a^**			**Finisher diet^b^**		
Corn, grain	60.01	59.89	59.77	58.83	68.80	69.35
Soybean meal (48%)^c^	33.47	33.49	33.51	22.50	25.74	25.30
Soybean oil	3.09	3.09	3.09	5.14	2.17	2.00
Defluorated phosphate	2.00	2.00	2.00	1.76	1.75	1.75
Limestone	0.68	0.68	0.68	0.65	0.65	0.65
Common salt	0.30	0.30	0.30	0.30	0.30	0.30
Vitamin premix^d^	0.25	0.25	0.25	0.25	0.25	0.25
Mineral premix^e^	0.08	0.08	0.08	0.08	0.08	0.08
AGP^f^	0.05	0.05	0.05	0.05	0.05	0.05
DL-methionine	0.02	0.12	0.22	0.00	0.01	0.12
Tryptophan	-	-	-	0.32	-	-
Threonine	-	-	-	1.00	0.06	-
L-Lysine HCL	-	-	-	1.50	0.08	0.10
Solka floc	-	-	-	7.57	-	-
Coccidiostat	0.05	0.05	0.05	0.05	0.05	0.05
Nutrient Content						
ME (kcal/kg)^g^	3100.00	3100.00	3100.00	3100.00	3100.00	3100.00
CP (%)^g^	21.00	21.00	21.00	18.00	18.00	18.00
SAA (%)^g^	0.70	0.80	0.90	0.52	0.62	0.72
Lysine				1.94	0.94	1.00
SAA:Lysine				0.27	0.66	0.72

a *DL-methionine was added at 0.02, 0.12 and 0.22% for 3 diets shown and remaining 3 diets were formulated as similar but without AGP.*

b *DL-methionine was added at 0, 0.01 and 0.12% for 3 diets shown and remaining 3 diets were formulated as similar but without AGP.*

c *Crude protein in parenthesis.*

d *Vitamin premix provided the following (per kg of diet): thiamin mononitrate, 2.4 mg; nicotinic acid, 44 mg; riboflavin, 4.4 mg; D-Ca pantothenate, 12 mg; vitamin B12 (cobalamin), 12.0 g; pyridoxine HCl, 4.7 mg; D-biotin, 0.11 mg; folic acid, 5.5 mg; menadione sodium bisulfite complex, 3.34 mg; choline chloride, 220 mg; cholecalciferol, 27.5 g; transretinyl acetate, 1,892 g; α tocopheryl acetate, 11 mg; ethoxyquin, 125 mg.*

e *Mineral premix provided the following (per kg of diet): manganese (MnSO4.H2O), 60 mg; iron (FeSO4.7H2O), 30 mg; zinc (ZnO), 50 mg; copper (CuSO4.5H2O), 5 mg; iodine (ethylene diamine dihydroiodide), 0.15 mg; selenium (NaSe03), 0.3 mg.*

f *BMD-50 (Bacitracin Methylene Disalicylate) was added as an antibiotic growth promoter.*

g *ME, metabolizable energy; CP, crude protein; SAA, sulfur amino acid.*

#### Measurement of Growth Performance and Carcass Yield

Feed intake, BWG, and FCR during both starter and finisher phases were determined as described in experiment I. The chicks and the leftover feed were weighed on days 21 and 42, to calculate total BWG, FI, and FCR. The FI and BWG of chickens were adjusted for mortalities. Mortality was recorded and transformed according to a previous study for analysis (24). At d 42, 5 chicks from each pen were selected and individually wing-banded for carcass yield evaluation. After 8 h of feed withdrawal, the chicks were weighed individually, slaughtered, and eviscerated in the processing plant at the University of Georgia. The carcasses were cleaned by removing feathers, feet, and visceral organs. The carcasses were chilled in slush ice overnight; the next day chilled carcass weights were measured, and yield was obtained for the entire carcass, pectoralis (P.) major, P. minor, whole thighs, and wings. The whole carcass was weighed first, then each respective organ was removed and weighed individually.

#### Measurement of Immune Genes and Amino Acid Transporter Genes by Quantitative Real-Time Polymerase Chain Reaction (qRT-PCR)

The jejunum and ileum samples were collected from one bird/replicate at 21 and 42 days for gene expression analysis. Briefly, total RNA from ileum and jejunum was extracted using the Qiazol reagent (Life Technologies, Valencia, CA, United States). The samples were then homogenized in a mini-bead beater-16 homogenizer (Biospec Products, Fisher Scientific, Bartlesville, OK) for 3 min. After extraction, the RNA pellets were dissolved in 200 μl nuclease-free water (Ambion, Applied BioSystems, Life Technologies, Carlsbad, CA, United States), and total RNA concentrations were determined at an optical density of 260 nm using a NanoDrop 2000 spectrophotometer (Thermo Fisher Scientific, MA, United States). The RNA was normalized to a concentration of 2μg/μl, and purity was verified at an optical density ratio of 260 to 280 nm. The normalized total RNA was then reverse-transcribed using high-capacity cDNA synthesis reverse transcription kits (Applied BioSystems, Life Technologies, Carlsbad, CA, United States) following the manufacturer's protocol. The synthesized cDNA was stored at −20°C. Quantitative real-time polymerase chain reaction (qRT-PCR) was performed in duplicate reaction including nuclease-free water, both forward and reverse primers, cDNA, and SYBR Green (Applied BioSystems, Life Technologies, Carlsbad, CA) The data were generated using the ΔΔCt method by normalizing the expression of the target gene to a housekeeping gene, Glyceraldehyde 3-phosphate dehydrogenase (GAPDH), and the values were reported as fold changes of the expression of the target genes in the experimental groups compared with the control group. The qRT-PCR was performed for immune genes including interleukin-1β (IL−1β), interferon-gamma (IFN-γ), interleukin-6 (IL-6), and interleukin (IL-10) for ileum, and transporter genes including the sodium-dependent neutral amino acid transporter B[0]AT1 (SLC6A19) and the large neutral amino acids transporters small subunit 1 (Lat1) for jejunum using a step one thermo-cycler (Applied Biosystems, Foster City, CA). Primers for genes of interest were designed and checked using GeneBank from the National Center for Biotechnology Information (NCBI) and are presented in Table 3.

**Table 3:** Information of primers for quantitative real-time PCR.

**Gene**	**Forward primer (5^′^-3^′^)**	**Reverse primer (3^′^-5^′^)**	**Annealing temperature °C**
GAPDH	GCTAAGGCTGTGGGGAAAGT	TCAGCAGCAGCCTTCACTAC	56
IL-1β	CACAGAGATGGCGTTCGTTC	GCAGATTGTGAGCATTGGGC	57
INF-γ	GCATCTCCTCTGAGACTGGC	GCTCTCGGTGTGACCTTTGT	57
IL-6	TTCGACGAGGCAAGGAACC	AGGTCTGAAAGGCGAACAGG	57
IL-10	GCTCTCCTTCCACCGAAACC	GGAGCAAAGCCATCAAGCAG	57
SLC6A19	TCTATTGAAGATTCGGGCAC	AATGGTAAGCACAAGGTATGG	56
LAT-1	TCCTTTTTACTTGTTTGGGGTC	GCTTTTCCTGCTTACGATTCT	54

*GADPH, Glyceraldehyde 3-phosphate dehydrogenase; IL-1β, Interlukin-1β; INF-γ, interferon-gamma; IL-6, Interleukin-6; IL-10, Interleukin-10; SLC6A19, sodium-dependent neutral amino acid transporter B(0)AT1; LAT-1, large neutral amino acids transporters small subunit 1*.

### Statistical Analysis

All data were analyzed by a 2 way-ANOVA, using the PROC GLM procedure of the SAS software (version 9.2) (SAS Institute Inc., Cary, NC). In both experiments, the main effects considered in the model were AGP, SAA, and their interaction. The significance of the differences among means was assessed by Tukey's multiple range test and *p* < 0.05 was defined as the level of significance.

## Results

### Experiment 1

From 0 to 7 d, there were no significant interactions (*p*>0.05) between the factors for FI, BWG, and FCR (Table 4). The BWG was affected by diets, in which birds fed 0.7% SAA had the lowest BWG (*p*<0.001). The FCR was affected by both AGP and SAA, and birds fed AGP had lower FCR than those not fed AGP (*p* = 0.022), and birds fed 0.9 and 1.1% SAA showed lower FCR than those fed 0.7% of SAA (*p* = 0.002). The FI was not significantly different between the treatments. From 8 to 14 d, a significant interaction was found (*p* = 0.045) for BWG. Birds fed 0.8% SAA showed higher BWG than those fed 0.7% in both with and without AGP groups. Increasing SAA levels higher than 0.8% tended to decrease BWG in birds not fed AGP while for birds fed AGP, such a trend was not observed as the SAA levels increased from 0.8 to 1.1%. During this phase, no interactions were found between FI and FCR. The FI was lower when birds were fed 0.7% SAA compared to 0.8%, and FCR was lower for birds fed 0.8, 0.9, and 1.0% SAA compared to 0.7% SAA (*p* < 0.02). From 15 to 21 d, no interactions between AGP and SAA were found, and the two factors did not affect the performance of the birds during this phase.

**Table 4:** Mean values of feed intake (FI), body weight (BW), and feed conversion ratio (FCR) of broilers fed different levels of sulfur amino acids (SAA) without or with an antibiotic growth promoter (AGP) in battery cages from 0 to 7, 7 to 14, and 14 to 21 d (experiment 1).

**AGP**	**SAA^2,3^**	**0–7d**				**8–14d**				**15–21d**				**0–21d**			
		**FI (kg)**	**BWG (kg)**	**BW (kg)**	**FCR**	**FI (kg)**	**BWG (kg)**	**BW (kg)**	**FCR**	**FI (kg)**	**BWG (kg)**	**BW (kg)**	**FCR**	**FI (kg)**	**BWG (kg)**	**BW (kg)**	**FCR**
-	1	0.128	0.104	0.149^b^	1.23	0.282	0.179^bcd^	0.297^bc^	1.57	0.478	0.290	0.587	1.69	0.888	0.573	0.587	1.49
	2	0.146	0.126	0.171^ab^	1.16	0.311	0.216^a^	0.353^a^	1.44	0.511	0.359	0.713	1.42	0.968	0.702	0.713	1.58
	3	0.152	0.136	0.180^a^	1.12	0.306	0.208^ab^	0.351^a^	1.47	0.484	0.315	0.666	1.53	0.942	0.657	0.666	1.50
	4	0.154	0.128	0.174^ab^	1.20	0.300	0.203^abc^	0.341^ab^	1.48	0.511	0.344	0.686	1.49	0.965	0.675	0.686	1.40
	5	0.141	0.126	0.179^a^	1.13	0.272	0.173^cd^	0.322^ab^	1.59	0.461	0.296	0.606	1.56	0.875	0.596	0.606	1.53
+	1	0.127	0.103	0.147^b^	1.22	0.278	0.157^d^	0.271^c^	1.77	0.523	0.303	0.574	1.74	0.929	0.563	0.574	1.43
	2	0.151	0.137	0.182^a^	1.11	0.304	0.201^abc^	0.347^a^	1.52	0.497	0.344	0.678	1.45	0.952	0.682	0.678	1.58
	3	0.140	0.138	0.181^a^	1.02	0.303	0.208^ab^	0.354^a^	1.46	0.525	0.363	0.717	1.45	0.968	0.708	0.717	1.37
	4	0.140	0.126	0.171^ab^	1.11	0.306	0.202^abc^	0.339^ab^	1.52	0.491	0.356	0.695	1.39	0.937	0.685	0.695	1.42
	5	0.149	0.134	0.179^a^	1.11	0.298	0.194^abc^	0.339^ab^	1.54	0.479	0.342	0.616	1.39	0.926	0.606	0.616	1.42
**AGP**																	
-		0.144	0.124	0.170	1.17^a^	0.294	0.196	0.333	1.51	0.489	0.321	0.651	1.61	0.927	0.641	0.651	1.50
+		0.141	0.128	0.172	1.11^b^	0.298	0.193	0.330	1.56	0.503	0.341	0.656	1.48	0.942	0.649	0.656	1.45
**SAA**																	
1		0.127	0.104^b^	0.148^b^	1.23^a^	0.280^b^	0.168	0.284^b^	1.67^a^	0.501	0.296	0.581^b^	1.71	0.908	0.568^b^	0.581^b^	1.46
2		0.149	0.131^a^	0.176^a^	1.13^ab^	0.307^a^	0.208	0.350^a^	1.48^b^	0.504	0.352	0.695^a^	1.44	0.960	0.692^a^	0.695^a^	1.58
3		0.146	0.137^a^	0.181^a^	1.07^b^	0.304^ab^	0.208	0.353^a^	1.47^b^	0.504	0.339	0.691^a^	1.59	0.955	0.683^a^	0.691^a^	1.44
4		0.147	0.127^a^	0.172^a^	1.16^ab^	0.303^ab^	0.202	0.340^a^	1.50^b^	0.501	0.350	0.690^a^	1.44	0.951	0.680^a^	0.690^a^	1.41
5		0.145	0.130^a^	0.179^a^	1.12^b^	0.285^ab^	0.184	0.330^a^	1.56^ab^	0.470	0.319	0.611^ab^	1.57	0.900	0.601^ab^	0.611^ab^	1.48
**P-Value**																
AGP		0.5600	0.3820	0.5881	0.0225	0.5647	0.5115	0.6441	0.1031	0.2584	0.1806	0.8474	0.2277	0.4288	0.7184	0.8474	0.3820
SAA		0.0563	<0.0001	<0.0001	0.0027	0.0272	<0.0001	<0.0001	0.0012	0.3566	0.1266	0.0060	0.4480	0.1480	0.0029	0.0060	0.3969
AGP*SAA		0.5264	0.8475	0.7941	0.6080	0.5264	0.0457	0.3727	0.1420	0.3113	0.6622	0.8238	0.6971	0.6054	0.8899	0.8238	0.9303
SE		0.0025	0.0024	0.0024	0.0136	0.0034	0.0032	0.0047	0.0191	0.0062	0.0078	0.0128	0.0526	0.045	0.061	0.0128	0.029

a−d *Means within a column with no common superscript differ significantly (p < 0.05).*

*Represent level of SAA in starter (1 = 0.7, 2 = 0.8, 3 = 0.9%, 4 = 1.0%, 5 = 1.1%).*

*SSA, sulfur amino acid.*

### Experiment 2

#### Growth Performance

From 0 to 21 d, no interactions were found between the factors for FI, BWG, and FCR (Table 5). The FI was the highest for birds fed 0.8% SAA (*p* = 0.040), the BWG was the lowest for birds fed 0.7% of SAA (*p* < 0.001), and the FCR was the lowest for birds fed 0.9%, followed by 0.8% of SAA, and birds fed 0.7% of SAA had the highest FCR (*p* < 0.001).

**Table 5:** Mean values of feed intake (FI), body weight (BW), body weight gain (BWG), and feed conversion ratio (FCR) of broiler chicks fed different levels of sulfur amino acids (SAA) without or with an antibiotic growth promoter (AGP) in floor pens from 0 to 21 and 22 to 42 d (*p* < 0.05) (experiment 2).

**AGP**	**SAA^2,3^**	**0–21d**					**22–42d**					**0–42d**				
		**FI (kg)**	**BWG (kg)**	**BW (kg)**	**FCR**	**Mortality^4^**	**FI (kg)**	**BWG (kg)**	**BW (kg)**	**FCR**	**Mortality**	**FI (kg)**	**BWG (kg)**	**BW (kg)**	**FCR**	**Mortality**
-	1	1.09	0.628	0.671^c^	1.74	1.013	2.06^b^	0.767^b^	1.481^c^	2.81	1.017	3.15^b^	1.395^c^	1.481^c^	2.30^a^	1.029
	2	1.16	0.746	0.788^ab^	1.56	1.007	2.04^b^	0.711^b^	1.440^c^	2.89	1.007	3.27^b^	1.457^c^	1.440^c^	2.27^a^	1.013
	3	1.11	0.772	0.814^a^	1.44	1.007	3.21^a^	1.44^a^	2.239^b^	2.26	1.000	4.32^a^	2.208^ab^	2.239^b^	1.97^ab^	1.007
+	1	1.13	0.667	0.708^bc^	1.71	1.010	3.33^a^	1.50^a^	2.303^ab^	2.24	1.000	4.46^a^	2.162^b^	2.303^ab^	2.07^ab^	1.010
	2	1.19	0.754	0.795^a^	1.58	1.003	3.14^a^	1.50^a^	2.342^ab^	2.07	1.010	4.52^a^	2.253^ab^	2.342^ab^	2.01^ab^	1.013
	3	1.13	0.791	0.833^a^	1.43	1.007	3.38^a^	1.69^a^	2.547^a^	1.99	1.007	4.53^a^	2.482^a^	2.547^a^	1.83^b^	1.013
**AGP**																
-		1.12	0.716	0.758	1.58	1.009	2.44	0.971	1.720	2.65^a^	1.008	3.58^b^	1.687^b^	1.720	2.179^a^	1.016
+		1.15	0.737	0.780	1.57	1.007	3.28	1.56	2.397	2.10^b^	1.006	4.50^a^	2.299^a^	2.397	1.969^b^	1.012
**SAA**																
1		1.11^b^	0.647^b^	0.690^b^	1.72^a^	1.012	2.70	1.131	1.892	2.52^a^	1.008	3.81^b^	1.788^b^	1.892	2.184^a^	1.020
2		1.18^a^	0.752^a^	0.792^a^	1.57^b^	1.005	2.59	1.105	1.891	2.48^ab^	1.008	3.89^b^	1.855^b^	1.891	2.139^a^	1.013
3		1.12^b^	0.782^a^	0.824^a^	1.43^c^	1.007	3.30	1.563	2.393	2.12^b^	1.003	4.43^a^	2.345^a^	2.393	1.898^b^	1.010
***P*** **-Value**																
AGP		0.1676	0.1711	0.1713	0.8577	0.5641	<0.0001	<0.0001	<0.0001	0.0002	0.4700	<0.0001	<0.0001	<0.0001	0.0034	0.3505
S		0.0404	<0.0001	<0.0001	<0.0001	0.3411	<0.0001	<0.0001	<0.0001	0.0326	0.3464	<0.0001	<0.0001	<0.0001	0.0027	0.2185
AGP*SAA		0.9476	0.7109	0.7264	0.8444	0.9187	<0.0001	0.0006	0.0002	0.2222	0.0114	< .0001	0.0009	0.0002	0.7158	0.0648
SE		0.0112	0.0122	0.0122	0.0264	0.0018	0.1036	0.0692	0.0777	0.0837	0.0018	0.1039	0.0746	0.0777	0.0415	0.0024

a−c *Means within a column with no common superscript differ significantly (p < 0.05).*

^2^ *Represent Level of SAA in Starter (1=0.7, 2=0.8 and 3=0.9%) and Finisher (1 = 0.52, 2 = 0.62, and 3 = 0.72%).*

^3^ *SSA, sulfur amino acid.*

^4^*Mortilty was transformed as $\sqrt{\frac{dead\;birds}{total\;birds}+1}$*.

From 22 to 42 d, interactions between SAA levels and AGP were found for FI and BWG (*p* < 0.001) (Table 5). Birds fed AGP showed higher FI and BWG than birds without AGP when receiving diets with 0.52 and 0.62% SAA, whereas birds fed 0.72 SAA showed similar results regardless of AGP addition in the diet. These results suggest that the highest level of SAA (0.72%) was able to reverse the poor results caused by the absence of AGP in the diet. The FCR was lower for birds fed AGP, and for birds fed 0.72% SAA compared to 0.52% SAA (*p* < 0.001, *p* = 0.032).

#### Carcass Yield

No interactions were found for the carcass yield parameters (*p* > 0.05). There was an effect of SAA levels on all parameters (*p* < 0.001) (Table 6). The birds fed 0.72% SAA had the highest chilled carcass weight, P. major weight, and P. minor weight, and the birds fed 0.62% SAA had intermediate value whereas the 0.52% SAA-fed birds had the lowest values. Both 0.72 and 0.62% SAA-fed birds had higher whole thigh and wings weight than the 0.52% SAA-fed birds. There was an effect of AGP on P. major where the AGP included groups that had higher P. major weight than the birds fed without AGP.

**Table 6:** Mean values of carcass yield of broiler finisher chicks fed different levels of sulfur amino acids (SAA) without or with an antibiotic growth promoter (AGP) on d 42 (*p* < 0.05) (experiment 2).

**AGP**	**SAA^**2, 3**^**	**Chilled carcass (kg)**	**P. major^**3**^ (g)**	**P. minor^**3**^ (g)**	**Whole thigh (g)**	**Wings (g)**
-	1	1.05^d^	183.7^d^	50.64^d^	343.5^b^	128.2^b^
	2	1.64^c^	345.5^c^	84.97^bc^	500.4^a^	188.3^a^
	3	1.80^ab^	399.5^ab^	111.4^a^	525.5^a^	195.8^a^
+	1	1.03^d^	196.0^d^	59.10^cd^	328.7^b^	125.2^b^
	2	1.72^bc^	370.5^bc^	91.17^ab^	522.5^a^	189.9^a^
	3	1.88^a^	439.5^a^	106.1^ab^	545.5^a^	201.0^a^
**AGP**						
-		149.8	309.6^b^	82.33	456.5	170.8
+		154.4	335.3^a^	85.47	465.9	172.0
**SAA**						
1		1.04^c^	189.8^c^	54.87^c^	336.1^b^	126.7^b^
2		1.68^b^	358.0^b^	88.07^b^	512.0^a^	189.1^a^
3		1.84^a^	419.5^a^	108.8^a^	535.5^a^	198.4^a^
**P-Value**						
AGP		0.0887	0.0150	0.5527	0.3230	0.7103
SAA		<0.0001	<0.0001	<0.0001	<0.0001	<0.0001
AGP*SAA		0.1512	0.5608	0.5224	0.1999	0.5954
SE		0.0291	0.0090	0.0031	0.0082	0.0029

a−d *Means within a column with no common superscript differ significantly (p < 0.05).*

^2^ *Represent Level of SAA in Finisher (1 = 0.52, 2 = 0.62, and 3 = 0.72%).*

^3^ *SAA, sulfur amino acid; P. major, Pectoralis Major; P. minor, Pectoralis minor.*

#### Immune Genes and Amino Acid Transporter Genes

At 21 d, there was a significant interaction between SAA and AGP for Lat1 gene expression in the jejunum of birds (*p* = 0.020) (Table 7). Birds fed 0.9% SAA and AGP showed higher expression of the LAT1 transporter compared to birds fed 0.70% SAA without AGP. No other significant differences were observed. The expression of IL-10 and IL−1β genes was upregulated in the ileum of birds fed AGP (*p* < 0.038). No differences between the treatments were found for SLC6A19, IL-6, and INF-γ expressions (*p* > 0.05).

**Table 7:** Mean values of relative expression of nutrient transporters in the jejunum and immune genes in the ileum and of chickens fed different levels of sulfur amino acids (SAA) without or with an antibiotic growth promoter (AGP) (*p*<0.05) (experiment 2).

**AGP**	**SAA^**2, 3**^**	**21d**						**42d**					
		**SLC6A19^**3**^**	**Lat1**	**IL-6**	**IL-10**	**IL-1β**	**INF-γ**	**SLC6A19**	**Lat1**	**IL-6**	**IL-10**	**IL-1β**	**INF-γ**
-	1	1.06	1.02^b^	1.01	1.08	1.27	1.07	1.02^a^	1.04	1.01	1.00	1.02	1.03
	2	0.785	1.52^ab^	1.14	2.21	1.61	1.57	0.320^b^	1.12	2.23	1.80	1.24	1.41
	3	1.32	1.28^ab^	1.02	1.21	1.68	1.90	0.310^b^	0.754	1.63	1.25	1.41	1.06
+	1	0.826	1.38^ab^	1.89	2.66	1.73	2.20	0.425^b^	1.27	1.45	1.30	1.28	1.55
	2	1.05	1.21^ab^	2.43	1.66	1.92	3.77	0.594^ab^	1.27	1.91	1.10	1.11	1.52
	3	1.05	1.69^a^	3.49	2.44	1.59	4.11	0.369^b^	1.04	1.54	1.13	1.28	1.08
**AGP**													
-		1.05	1.27	1.52	1.05^b^	1.50^b^	1.52	0.550	0.972	1.22	1.51^b^	1.35	1.22
+		0.974	1.42	1.74	2.60^a^	2.25^a^	1.74	0.462	1.19	1.22	3.36^a^	1.18	1.22
**SAA**													
1		0.945	1.20	1.50	1.45	1.87	1.50	0.722	1.15	1.15	1.64	1.15	1.15
2		0.916	1.36	1.76	1.78	1.94	1.76	0.457	1.20	1.17	2.67	1.45	1.17
3		1.18	1.48	1.63	2.25	1.83	1.63	0.339	0.896	1.35	3.00	1.19	1.35
***P*** **-Value**													
AGP		0.5942	0.1626	0.4966	0.0005	0.0382	0.4966	0.3346	0.2391	0.9985	0.0016	0.4484	0.9985
SAA		0.3008	0.1165	0.8042	0.2193	0.9649	0.8042	0.0108	0.3612	0.8882	0.0785	0.5024	0.8882
AGP*SAA		0.2875	0.0200	0.7850	0.1999	0.0425	0.7850	0.0031	0.9525	0.8544	0.5721	0.2080	0.8544
SE		0.0750	0.0655	0.2475	0.1909	0.1461	0.3193	0.0571	0.0855	0.1647	0.1138	0.1420	0.1107

ab *means within a column with different superscripts trend to be different (P < 0.05).*

^2^Represent Level of SAA in Starter (1 = 0.7, 2 = 0.8, and 3 = 0.9%) and Finisher (1 = 0.52, 2 = 0.62, and 3 = 0.72%).

^3^*SAA, sulfur amino acid; SLC6A19, sodium-dependent neutral amino acid transporter B(0)AT1; Lat-1, large neutral amino acids transporters small subunit 1; IL-6, Interleukin-6; IL-10, Interleukin-10; IL-1β, Interlukin-1β; INF-γ, interferon-gamma*.

At 42 d, there was a significant interaction between SAA and AGP for SLC6A19 expression in the jejunum (*p* = 0.03). The birds fed 0.7% SAA without AGP had higher expression of this gene than those fed 0.7% with AGP, 0.8% without AGP, and 0.9% with and without AGP. The IL-10 expression was upregulated in the ileum when birds were fed AGP (*p* = 0.001), and no differences were found between the treatments for the expression of Lat1 in the jejunum and IL-6, IL−1β and INF-γ in the ileum (*p* > 0.05).

## Discussion

Determination of the essential nutrients such as SAA with/without AGP has not been investigated despite studies that have tested AGP alternative compounds supplementation against AGP and how they affected optimal growth performance, immunity, and gut microbiome in poultry (25, 26). We compared different levels of SAA with/without supplementation of AGP and observed interactions between these two factors in growth performance and key gene expression of immunity and nutrient transport. It was reported that supplementation of AGP and sulfaquinoxaline to chicks from 0 to 21 d resulted in better FCR than in unmedicated chicks, but there was no any interaction between the increasing levels of methionine and AGP (13). However, bacitracin improved FCR and BWG in these studies. The estimates of SAA requirements for broiler chicks from 3 to 6 weeks of age ranged from 0.72 to 0.80% (27). Our inclusions of 0.72 and 0.80% SAA to the diets for chickens from 3 to 6 weeks of age were in the range of the study by Hickling *et al*. (14). Mendonca and Jensen (28) conducted a study with a corn–soybean meal diet (3,200 kcal/kg of ME) supplemented with DL-methionine and reported that 0.78% SAA was needed to maximize feed efficiency/FCR. However, our FCR values for finisher chicks were like Lei, Ru (26), where they reported FCR of 1.99 obtained with AGP. In our finisher diet, we chose 3 levels of SAA, and 0.72% SAA (SAA to Lys = 0.72%), which was the highest level, with and without AGP showed the best growth performance compared to the other levels (0.27 and 0.66% SAA to Lys ratios). These results suggest that there has not been a drastic change in the requirement of SAA when we compared our values with previous works discussed above. After 21 d, the addition of AGP in finisher diets with the highest SAA level had significantly positive effects on BWG, which was like a study reported by Lei, Ru (26). In a study by Matsushita, Takahashi (29), diets were formulated with salinomycin and enromycin as antibacterial agents. We used 0.05% bacitracin as an AGP source in both of our studies unlike in a study by Lei, Ru (26) that used only 0.02%.

Factors such as environment, management, and nutrition can affect the outcome of broiler feeding studies. Yang, Iji (30) discussed some of the factors including environment, management, nutrition, bird type, age, and stage of production that would lead to contrasting results in studies with probiotics. This can be applied to the effects of AGP in our study. The battery cages used in Exp. 1 had a rather cleaner facility where droppings were collected directly below the raised wires and hygiene was maintained throughout for 21 d. The shavings initially used in our floor pen study were clean and as the chickens grew up until d 42, the shavings were not replaced and were contaminated with droppings of chicks. We cannot maintain chickens in battery cages (Experiment 1) for more than 21 d due to their increasing body weight and size. In this study, when chickens were raised beyond 21 d in the floor pen, there was more probability of chickens getting susceptible to germs, and we had observed a stronger effect of AGP on growth performances.

The pattern of *P. major* yield obtained from our study was similar to the value reported by Matsushita, Takahashi (29), where the AGP-added diet showed a higher yield only in *P. major*, but there was no greater difference in the rest of the variables. The addition of DL-methionine at 0.15% in a finisher chick diet without AGP resulted in a similar yield for *P. major* (31). The whole thigh yield from diets containing 0.72% SAA in our study was >0.78% SAA with AGP reported by Goulart, Costa (32).

The role of methionine and cysteine in enhancing immune function has been well described (33). However, an increased methionine content, above the level required for optimal growth, improves the immune responses (3). The previous studies have shown that under stressed conditions, methionine supplementation can alter the expression of pro- or anti-inflammatory cytokines (34, 35). However, such alteration in expressions was not observed in this study possibly because the birds were raised in normal conditions.

Interleukin-1β is a pro-inflammatory cytokine secreted from monocytes and macrophages and mediates inflammatory response (36). The IL-1β activates a range of cells including macrophages and T lymphocytes that may thus lead to the production of other cytokines and chemokines. Similar to our study, a higher mRNA expression of IL-1β was observed in deccox and monensin-treated group compared to the untreated group in the study by Lee et al. (22). In this study, the higher IL-10 expression level in AGP-supplemented diets indicated that there was a suppression of inflammation because IL-10 was an anti-inflammatory cytokine. However, in the finisher phase, we did not see any differences in the expressions of all the immune cytokines tested between the groups with and without AGP.

Dietary regulation of intestinal amino acid (AA) transporter genes has been studied for several amino acids (37). Amino acids are transported across the plasma membrane by different transporter systems (38). Met and Cys are neutral AA which are transported by the common neutral AA transporters such as solute carrier family 6 member 19 (SLC6A19), L- type AA transporter (LAT-1), and LAT-4 (39, 40). A previous study has found that methionine-deficient diets would lead to downregulation of LAT1 in the broilers which correspond to the result found in this study that broilers fed lower SAA diets showed lower expression of LAT1. However, the SLC6A19 expression levels decreased as the SAA levels increased as pointed out by previous research that higher expression of AA transporter shown in the intestinal tissue correlates with the phenomenon to overcome the lower concentration of SAA that reaches the specific section of the intestine (41). The results also indicated that the effects of these two transporters are regulated differently by the SAA levels in the diets. Besides, the effects of SAA levels on expressions of AA transporters were influenced by the presence of AGP because a significant interaction effect was observed in this study. The mechanism that led to these findings would require further study.

## Conclusion

Feeding broilers with diets supplemented with SAA at previously determined levels (0.88–0.90% for starter and 0.74% for finisher) was the best for growth performance. AGP-supplemented birds had better FCR and immune status, suggesting that AGP can be supplemented in finisher diets because of the increasing challenge (e.g., poor hygiene) in the floor pen environment. Broilers reared in an environment containing ubiquitous microorganisms may have a constant activation of the immune response. More studies should be conducted with different pathogen challenge models to find the relationships between total sulfur amino acids and immunity in broilers.

## Data Availability Statement

The raw data supporting the conclusions of this article will be made available by the authors, without undue reservation.

## Ethics Statement

The animal study was reviewed and approved by the University of Georgia Institutional Animal Care and Use Committee.

## Author Contributions

PA and WK: conceptualization. PA and FC: methodology, data collection, and formal analysis. PA, FC, and GL: writing. WK: writing—review and editing. All authors have read and agreed to the published version of the manuscript.

## Conflict of Interest

The authors declare that the research was conducted in the absence of any commercial or financial relationships that could be construed as a potential conflict of interest.

## Publisher's Note

All claims expressed in this article are solely those of the authors and do not necessarily represent those of their affiliated organizations, or those of the publisher, the editors and the reviewers. Any product that may be evaluated in this article, or claim that may be made by its manufacturer, is not guaranteed or endorsed by the publisher.
